# Evaluation of The Antioxidant, Antimicrobial, and Anticancer Activities of *Dicliptera bupleuroides* Isolated Compounds Using In Vitro and In Silico Studies

**DOI:** 10.3390/molecules26237196

**Published:** 2021-11-27

**Authors:** Shehla Akbar, Saiqa Ishtiaq, Muhammad Jahangir, Sameh S. Elhady, Hanin A. Bogari, Abdelrahman M. Alahdal, Mohamed L. Ashour, Fadia S. Youssef

**Affiliations:** 1 Department of Pharmacy, Punjab University College of Pharmacy, University of the Punjab, Lahore 05422, Pakistan; 2 Department of Chemistry, Government College University, Lahore 54000, Pakistan; mjahangir.gcu@gmail.com; 3 Department of Natural Products, Faculty of Pharmacy, King Abdulaziz University, Jeddah 21589, Saudi Arabia; ssahmed@kau.edu.sa; 4 Department of Pharmacy Practice, Faculty of Pharmacy, King Abdulaziz University, Jeddah 21589, Saudi Arabia; hbogari@kau.edu.sa (H.A.B.); aalahdal2@hotmail.com (A.M.A.); 5 Pharmacy Program, Department of Pharmaceutical Sciences, Batterjee Medical College, Jeddah 21442, Saudi Arabia; 6 Department of Pharmacognosy, Faculty of Pharmacy, Ain-Shams University, Abbasia, Cairo 11566, Egypt; fadiayoussef@pharma.asu.edu.eg

**Keywords:** acanthaceae, antioxidant activity, anticancer activity, antimicrobial activity, *Dicliptera bupleuroides*, molecular modeling, phytoconstituents

## Abstract

Phytochemical investigation of chloroform fraction (DBC) and ethyl acetate fraction (DBE) of *D. bupleuroides* (Acanthaceae) resulted in the isolation of β-sitosterol (**1**) from DBC and vanillic acid (**2**) from DBE, which were first to be isolated from *D. bupleuroides.* β-Sitosterol (**1**) exhibited substantial antioxidant activity (IC_50_ = 198.87 µg/mL), whereas vanillic acid (**2**) showed significant antioxidant power (IC_50_ = 92.68 µg/mL) employing 1,1-diphenyl-2-picrylhydrazyl (DPPH*) radical scavenging capacity assay. Both compounds showed pronounced antimicrobial activity using the agar disc diffusion method, particularly against fungi showing MIC values of 0.182 and 0.02 concerning *Candida albicans,* respectively, and 0.001 mg/mL regarding *Penicillium notatum.* They revealed considerable antibacterial activity with MIC values ranging between 0.467 and 0.809 mg/mL. Vanillic acid (**2**) exhibited substantial anticancer potential displaying 48.67% cell viability at a concentration of 100 μg/mL using MTT (3-(4,5-dimethylthiazol-2-yl)-2,5-Diphenyl-2H-Tetrazolium Bromide) assay concerning HepG2 cell lines. These results were further consolidated by in silico studies on different enzymes, where vanillic acid displayed a high fitting score in the active pockets of DNA-gyrase, dihydrofolate reductase, aminoglycoside nucleotidyltransferase, and β-lactamase. It also inhibited human cyclin-dependent kinase 2 (CDK-2) and DNA topoisomerase II, as revealed by the in silico studies. ADME/TOPKAT (absorption, distribution, metabolism, excretion, and toxicity) prediction showed that vanillic acid exhibited reasonable pharmacodynamic, pharmacokinetic, and toxicity properties and, thus, could perfectly together with *D. bupleuroides* crude extract be incorporated in pharmaceutical preparations to counteract cancer and microbial invasion, as well as oxidative stress. Thus, it is concluded that *D. bupleroides* could be a potential source of therapeutically active compounds, which would be helpful for the discovery of clinically effective and safe drugs.

## 1. Introduction

Medicinal plants have been used since ancient times for the benefit and welfare of human beings. As time passed, pharmaceutical industries started to use medicinal plants to manufacture herbal preparations based on established therapeutic efficacy explored from crude extracts or their essential oils [1,2]. Herbal medicine is widely used by different communities all over the globe as it is simple, cheap, and has fewer side effects compared to medicines of synthetic origin [3,4]. The considerable effectiveness of herbal medicine in alleviating various serious ailments has mainly relied upon phytoconstituents, especially the secondary metabolites, such as flavonoids, alkaloids, tannins, anthraquinone derivatives, glycosides, and other phenolic compounds [5]. Today, almost 30% of the pharmaceutical preparations are based on plants, and it is highly observable that most developed countries import their raw materials of therapeutically important plants from developing countries [6].

Cancer and infectious diseases due to bacteria, fungi, and viruses are regarded as the most detrimental diseases in the current era all over the globe. Despite the enormous progress in the medicinal strategies for the curing of many human health problems, they still constitute a major impendence to public health and are considered as leading causes of morbidity and mortality. Although chemotherapeutic agents can alleviate symptoms, prolong life, or cause complete cure in some cancer cases, lack of selectivity is the most deleterious problem facing chemotherapy causing massive damage to both cancer, as well as normal, cells. For the infectious diseases, lack of medicine, in addition to the appearance of vigorous drug resistance, causes their dissemination in developing countries. Therefore, the need for anticancer and anti-infective drugs from natural resources to act as promising lead candidates in future drug discovery programs has been recently recognized to be obligatory worldwide. Plant kingdom represents an appealing, unique, and diverse resource. It furnishes a vast array of bioactive secondary metabolites [7,8].

The *Acanthaceae* family includes about 250 genera and nearly 2500 species belonging to dicotyledonous flowering plants, whereas most of them are shrubs and tropical herbs [9]. Traditionally, members of the family *Acanthaceae* were adopted to treat wounds externally; they play a crucial role in treating various lethal diseases, acting as an antioxidant, antipyretic, cytotoxic, antifungal insecticidal, immunomodulatory, and hepatoprotective, as well as antiviral, agent [10]. These promising activities are due to the presence of many secondary metabolites represented by naphthoquinone, benzenoids, flavonoids, triterpenoids, and glycosides. Local people of kadyala demonstrated that they used the fresh leaves of this plant to cure diabetes, poultice in eczema, juice to cure stomach troubles, as a tonic, in eye diseases, in treatment of GIT (gastrointestinal tract) problems, in cough, in fever, and inflammation of wounds [11,12,13,14]

*Dicliptera bupleuroides* is a perennial flowering herb that belongs to the *Acanthaceae* family. *Dicliptera bupleuroides* Nees is the new name of *Dicliptera roxburghiana* Nees according to Nees taxonomical classification and is commonly known as Kirch or Kalu or Kaali boti [15]. It is characterized by ovate leaves with acuminate margins with linear branches and hairy twigs that reach 90 cm in length. Traditionally, it is prevalent among different tribes by its effectiveness in curing snakebite wounds, stomach disorders, and bone fractures. Besides, it revealed considerable antimicrobial, antioxidant, and hepatoprotective potential, in addition to anthelmintic gastro-protective and insecticidal activities. This reputation is undoubtedly due to its richness in flavonoids, phenolic compounds, lipids, ascorbic acid, glycosides, and starch.

Thus, in this study, we aimed to perform a phytochemical investigation of *Dicliptera bupleuroides* whole plant to isolate its major compounds using different chromatographic techniques. These compounds were structurally elucidated via different spectroscopic techniques, such as NMR and IR. Consequently, the isolated compounds were subjected to in vitro biological investigations to evaluate their antioxidant, antimicrobial, and cytotoxic effect. The former was performed using 2,2-diphenyl-1-picrylhydrazyl scavenging capacity; meanwhile, the antimicrobial activity was assessed versus a panel of bacteria and fungi using disc diffusion assay. However, the cytotoxic effect was evaluated using the MTT assay. To further consolidate the obtained in vitro results, molecular modeling was performed for the isolated compounds on different proteins crucial in the occurrence and dissemination of microbial infection and cancerous cells. Additionally, the determination of ADMET (absorption, distribution, metabolism, excretion, and toxicity) properties and TOPKAT (toxicity prediction) of the isolated molecules was performed using Discovery Studio 4.5 software (Accelrys Inc., San Diego, CA, USA) to foresees better their possible pharmacokinetic, pharmacokinetic, and toxicity properties.

## 2. Results and Discussion

### 2.1. Phytochemical Characterization

**Figure 1:**
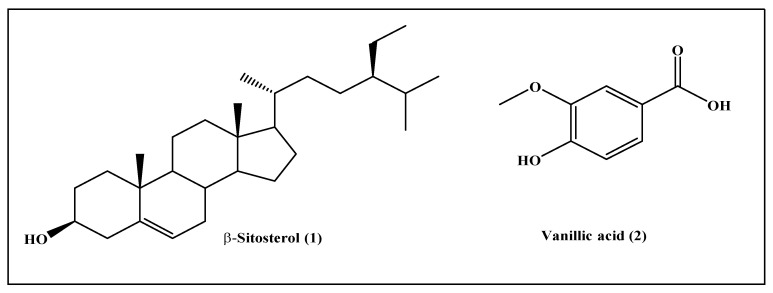
Compounds isolated from *D. bupleuroides* chloroform and ethyl acetate fractions.

Phytochemical investigation of chloroform fraction (DBC) and ethyl acetate fraction (DBE) of *D. bupleuroides* resulted in the isolation of two known compounds. Their structures were elucidated entirely via comparing their 1D and IR data with previously reported data existing in the literature. They were identified as β-sitosterol (**1**) from DBC [16,17,18] and vanillic acid (**2**) from DBE [19,20,21], which were first to be isolated from *D. bupleuroides* (Figure 1).

Compound (**1**) was collected as white needle-like crystals with a melting point of 138 °C. The IR spectrum showed absorption bands at different peaks (Figure S1, Supplementary Materials), whereas the ^1^H-NMR spectral data showed the presence of seventy hydrogen that are attached at CH_3_, CH_2_, CH, and OH groups. The appearance of singlet at δ_H_ of 0.98, 1.16, and 1.23 ppm confirmed the presence of three CH_3_ attached to quaternary carbons; meanwhile, the multiplets that appeared at δ_H_ of 0.78 and 0.90 ppm indicated that different CH_2_ groups attached with neighbor OH groups. The multiplets that appeared at δ_H_ 2.34 ppm are due to the attachment of carbon with the OH group. Overlapping signals of triplet at δ_H_ 5.34 ppm indicated the presence of one olefinic proton. The DEPT-90 spectrum presented the existence of 30 carbons. These carbons could be classified as CH_3_, CH_2_, CH, or quaternary carbons. The recognizable signal at δ_C_ of 130.59 and 121.75 ppm is assigned to the double bond between carbon atoms at positions 5 and 6, respectively. The signal at δ_C_ 71.85 ppm is assigned for C3 β-OH group; however, additional further signals at δ_C_ of 11.86 and 19.82 ppm are due to methyl carbons at C19 and C18, respectively. DEPT-135 indicated upward peaks due to CH_3_ and CH, while downward peaks are attributed to the presence of CH_2_ groups. Based on the physical properties (colorless needle-like crystals, melting point, and steroid test), spectroscopic analysis (IR and NMR), and the data obtained from the previous scientific literature, the isolated compound is identified as β-sitosterol [16,17,18]. ^1^H-NMR and ^13^C-NMR and IR spectral data of compound (**1**) are represented as Figure S1 in the Supplementary Materials.

Compound (**2**) was obtained as a white solid with a melting point equals 210–213 °C. The IR spectrum showed absorption bands at different peaks (Figure S2). The values indicated the presence of asymmetrical CH3 deformation found at a wavenumber of 1594 and 1521 cm^−1^ and symmetrical deformation existing at 1472–1376 cm^−1^. The presence of the O–CH3 group is confirmed by stretching vibration at 2836 cm^−1^. The strong peak at wavenumber below 900 cm^−1^ indicated the aromatic nature; meanwhile, O–H showed different vibrations between 800–700 cm^−1^ [19]. ^1^H-NMR (500 MHz, CDCl_3_) revealed peaks at δ_H_ (ppm): 7.70 (1H, dd, *J* = 1.5, 2.0 Hz, H-2), 7.57 (1H, d, *J* = 2.0 Hz, H-6), 6.04 (1H, s, H-5), 3.94 (3H, s, OCH_3_) that further confirmed the aromatic nature of the compound [20,21], thus, compound (**2**) was identified as vanillic acid (**2**). ^1^H-NMR and IR spectral data of compound (**2**) are represented as in Figure S2 in the Supplementary Materials.

### 2.2. In Vitro Biological Evaluation of D. bupleuroides Isolated Compounds

#### 2.2.1. In Vitro Antioxidant Activity Evaluation Using 1,1-Diphenyl-2-Picrylhydrazyl (DPPH*) Radical Scavenging Capacity Assay

The free radical theory has led to the search of compounds with an antioxidant activity in order to improve physiological conditions, such as delaying aging. In general, the starting point is based on the screening of different compounds using in vitro tests, such as the DPPH assay. This is the method most frequently used as it is rapid, simple, inexpensive, and robust and can be monitored over time, providing an idea of the steady state of the compound. The antioxidant activity can be analyzed by different methods both in vitro and in vivo. Among in vitro assays, the DPPH method is probably the most popular one due to its simplicity, speed, and low cost [22]. This assay relied upon the bleaching of the purple color of DPPH methanol solution in the presence of electron or hydrogen atom molecules that result in a decrease in the amount of free radicles and is related directly to the antioxidant capability of the examined sample [23]. Both isolated compounds, β-sitosterol (**1**) and vanillic acid (**2**), were examined for their antioxidant potential and compared to ascorbic acid, a standard antioxidant drug, using 1,1-diphenyl-2-picrylhydrazyl (DPPH*) radical scavenging capacity assay. Results illustrated in Table 1 revealed that β-sitosterol (**1**) exhibited significant antioxidant power displaying IC_50_ value of 198.87 µg/mL. However, vanillic acid (**2**) showed significant antioxidant power manifested by its IC_50_ value that is equal to 92.68 µg/mL. Vanillic acid (**2**) exceeds the antioxidant power of ascorbic acid that revealed an IC_50_ value of 125.86 µg/mL. This activity followed the previously published data about vanillic acid, a phenolic compound existing in edible plants, mainly fruits. It was previously reported to possess a wide array of biological activities represented mainly by antioxidant potential via scavenging free radicals, in addition to chemo-preventive effects [24]. It also showed in vivo antioxidant activity manifested by its high effectiveness on lipid peroxidation evidenced by reduction of malondialdehyde (MDA) with concomitant stimulation of endogenous antioxidant enzymes, including the elevation of catalase (CAT), glutathione peroxidase (GPx), superoxide dismutase (SOD) and total antioxidant capacity (TAC) in the rat hearts that exposed to Ischemia-reperfusion [25]. However, β-sitosterol revealed antioxidant activity in many studies via scavenging free radicals [26]. An additional study showed a potent in vivo antioxidant potential via increasing both enzymatic and non-enzymatic antioxidants in 1,2-dimethylhydrazine-induced colon carcinogenesis in rats. It enhanced the antioxidant enzymes via stimulating the estrogen receptor/PI3 kinase-dependent pathway. Besides, it scavenges the reactive oxygen species, evidenced by restoring GSH and GSH/total glutathione ratio upon treatment with β-sitosterol [27].

#### 2.2.2. In Vitro Antimicrobial Activity Evaluation Using Disc Diffusion Assay

Both compounds β-sitosterol (**1**) and vanillic acid (**2**) were examined for their antimicrobial activity versus a panel of bacteria and fungi and compared to standard antimicrobial agents, using the disc diffusion method. These organisms include *Staphylococcus aureus* (ATCC 6538), *Bacillus substilis* (ATCC 6633), *Echercheria coli* (ATCC 8739), *Pseudomonas aeruginosa* (ATCC 9027), and fungal strains *Candida albicans* (ATCC 10231) and *Penicillium notatum* (ATCC 11709). MIC values were calculated compared to commonly used antibiotics, namely ciprofloxacin for bacteria and fluconazole for fungi. Results illustrated in Table 2 revealed that both β-sitosterol (**1**) and vanillic acid (**2**) showed pronounced antimicrobial activity, particularly against fungi, showing MIC values of 0.182 and 0.02 mg/mL versus *C. albicans,* respectively, and 0.001 mg/mL versus *P. notatum.* It exceeds in this approach fluconazole, the standard antifungal agent that revealed 0.12 and 0.02 mg/mL versus the two fungi, respectively. Meanwhile, β-sitosterol (**1**) and vanillic acid (**2**) revealed considerable antibacterial activity, with MIC values range between 0.467 and 0.809 mg/mL, where *B. subtilis* is the most susceptible to the inhibitory potential of β-sitosterol (**1**) and vanillic acid (**2**), displaying MIC of 0.599 and 0.467 mg/mL, respectively (Table 2).

Additionally, vanillic acid showed potent antimicrobial activity in many recent studies. It acts as a flavoring agent in many edible plants and fruits and is highly recommended as a preservative. It has recently proved to destroy the integrity of the cell membrane of carbapenem-resistant *Enterobacter cloacae,* evidenced by a pronounced reduction in the intracellular ATP concentration, membrane potential, and pH with concomitant alteration in the morphology of the cell. Besides, it effectively inhibited carbapenem-resistant *Enterobacter cloacae* biofilm formation, as evidenced by confocal laser scanning microscopy, field emission scanning electron microscopy, and crystal violet staining [28]. Moreover, some previous studies on β-sitosterol revealed its antimicrobial potential; however, further in-depth mechanistic investigations must be conducted to consolidate the claimed antimicrobial activity [29]. Meanwhile, a recent study performed on β-sitosterol revealed the ability of β-sitosterol to prevent peptidoglycan biosynthesis, as well as bacterial cell wall formation via prevention of MurA and SrtA, is the main factor of the antibacterial activity of β-sitosterol, as revealed from molecular modeling [30].

#### 2.2.3. In Vitro Anticancer Activity Evaluation Using MTT (3-(4,5-dimethylthiazol-2-yl)-2,5-Diphenyl-2H-Tetrazolium Bromide) Assay

**Figure 2:**
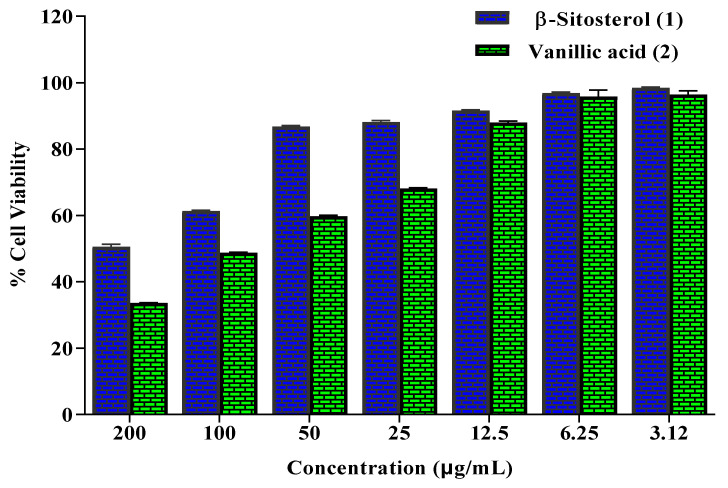
Percentage cell viability of HepG2 cells in MTT assay exhibited by β-sitosterol (**1**) and vanillic acid (**2**) isolated from *D. bupleuroides*.

The MTT assay is a colorimetric method that is used to evaluate the cell metabolic activity. Upon exposure of NAD(P)H-dependent cellular oxidoreductase enzymes to certain conditions, they reflect the number of viable cells present. These enzymes are able to reduce the tetrazolium dye MTT 3-(4,5-dimethylthiazol-2-yl)-2,5-diphenyltetrazolium bromide to its insoluble formazan, which has a purple color [31]. β-Sitosterol (**1**) and vanillic acid (**2**) isolated from *D. bupleuroides* were examined for their anticancer activity against HepG2 cell lines using MTT (3-(4,5-dimethylthiazol-2-yl)- 2,5-Diphenyl-2H-Tetrazolium Bromide) assay employing different concentrations. Results displayed in Figure 2 indicated that vanillic acid (**2**) at different concentrations exhibited substantial inhibition to cell viability that further demonstrated its substantial anticancer potential displaying 48.67% cell viability at 100 μg/mL. However, β-sitosterol (**1**) exerted weak anticancer activity, displaying 50.49% cell viability at 200 μg/mL. Meanwhile, DMSO and control revealed 100% cell viability, whereas doxorubicin, a standard anticancer agent, showed no cell viability at 33.3 μg/mL. Vanillic acid was previously reported to possess anticancer potential in many studies that were further consolidated by tracing its probable mode of action where vanillic acid prevents the synthesis of hypoxia-inducible factor 1 (HIF-1) that is significantly crucial in the adaptation of tumor to microenvironmental hypoxia. It effectively suppressed the expression of apamycin/p70 ribosomal protein S6 kinase/eukaryotic initiation factor 4E-binding protein-1 and Raf/extracellular signal-regulated kinase (ERK) kinase (MEK)/ERK pathways that consequently prohibited HIF-1α expression. Besides, it prohibited the expression of both VEGF and EPO proteins in a dose-dependent manner that further reflected on the prevention of angiogenesis. Vanillic acid considerably causes the induction of G1 phase arrest, in addition to inhibition of human colon cancer HCT116 cells, that was further supported by in vivo study in a xenografted tumor model [32].

### 2.3. In Silico Studies

#### 2.3.1. Molecular Docking Experiments

Molecular docking was performed for β-sitosterol (**1**) and vanillic acid (**2**) using Discovery Studio 4.5 (Accelrys Inc., San Diego, CA, USA) with C-docker protocol on different proteins that are crucial in the occurrence and dissemination of microbial infection and cancerous cells. Vanillic acid (**2**) exhibited the highest fitting versus all of the examined enzymes, showing high docking scores approaching that of the co-crystalized ligands and even exceeding them in its fitting within the active sites of some examined enzymes.

Regarding microbial enzymes, vanillic acid displayed a high fitting score in the active pockets of all examined enzymes showing binding energies (∆G) of −24.26, −26.63, −25.21, and −25.10 kcal/mole within the active centers of DNA-gyrase, dihydrofolate reductase, aminoglycoside nucleotidyltransferase, and β-lactamase, respectively, exceeding DNA-gyrase co-crystalized ligand. The co-crystalized ligands displayed binding energies (∆G) of −9.7, −28.90, −20.03, and −61.76 kcal/mole in DNA-gyrase, dihydrofolate reductase, aminoglycoside nucleotidyltransferase, and β-lactamase, respectively (Table 3).

**Figure 3:**
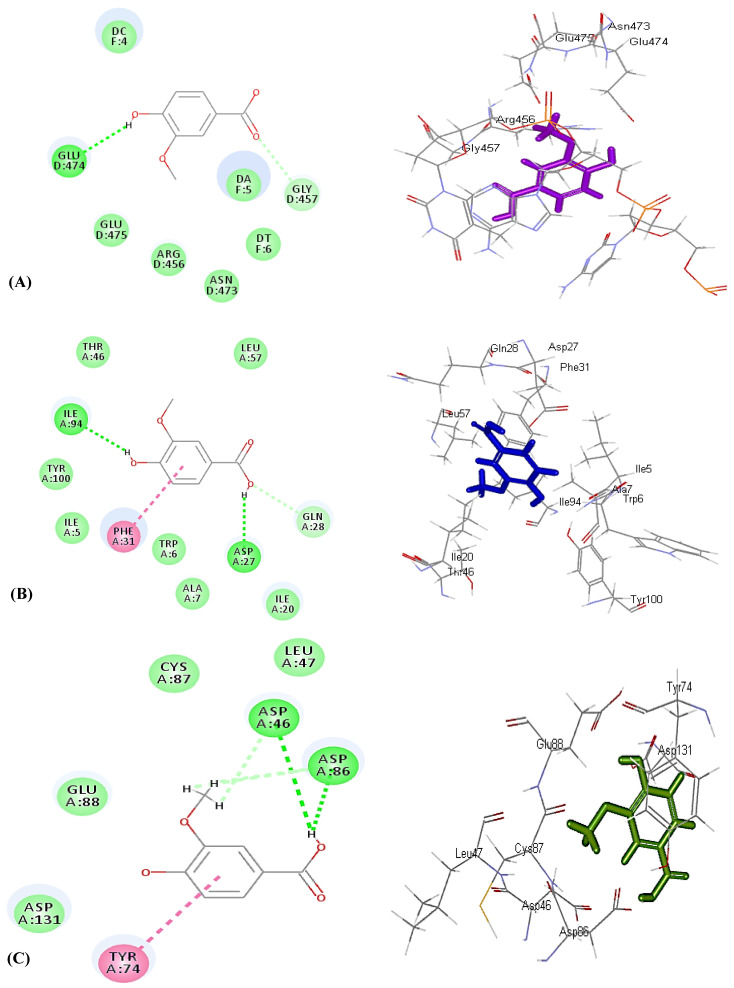
Two-dimensional and 3D binding mode of vanillic acid (**2**) within the active site of DNA-gyrase (**A**), dihydrofolate reductase (**B**), and Aminoglycoside nucleotidyltransferase (**C**).

The tight-fitting of vanillic acid in the active center of enzymes can be interpreted in virtue of the formation of multiple bonds with the amino acid residues at the active sites, where it forms one conventional H-bond with Glu474, in addition to one C–H bond with Gly457 in the case of DNA-gyrase (Figure 3A). Meanwhile, it forms two conventional H–bonds with Ile94 and Asp27, in addition to the formation of one π–π bond with Phe31 with dihydrofolate reductase (Figure 3B). Regarding aminoglycoside nucleotidyltransferase, it forms two H–bonds and two C–H bonds with Asp46 and Asp86, in addition to one π–π bond with Tyr74 (Figure 3C). In contrast, it forms three H–bonds with Ile117, Lys87, and Ser84, one π–alkyl bond with Ile117, and two C–H bonds Tyr141 and Gly144 with β-lactamase (Figure S3, Supplementary Materials). Thus, from the results of in silico studies, it can be concluded that vanillic acid exerted its antimicrobial activity via different mechanisms as it effectively inhibited DNA-gyrase that controls the supercoiling of microbial DNA, prohibiting dihydrofolate reductase that catalyzes NADPH-dependent reduction of dihydrofolate to tetrahydrofolate that is required for the production of microbial proteins and nucleic acids and, thus, its prohibition of leads to disruption in DNA synthesis causing ultimate cell death [33,34]. Furthermore, vanillic acid effectively inhibited β-lactamase to which bacterial resistance is attributed [35], in addition to the prohibition of aminoglycoside nucleotidyltransferase that is involved in bacterial resistance to aminoglycoside antibiotics that alters the structure of the antibiotics via adenylation and, hence, inactivates the drug, resulting in its deactivation towards bacteria [36].

**Figure 4:**
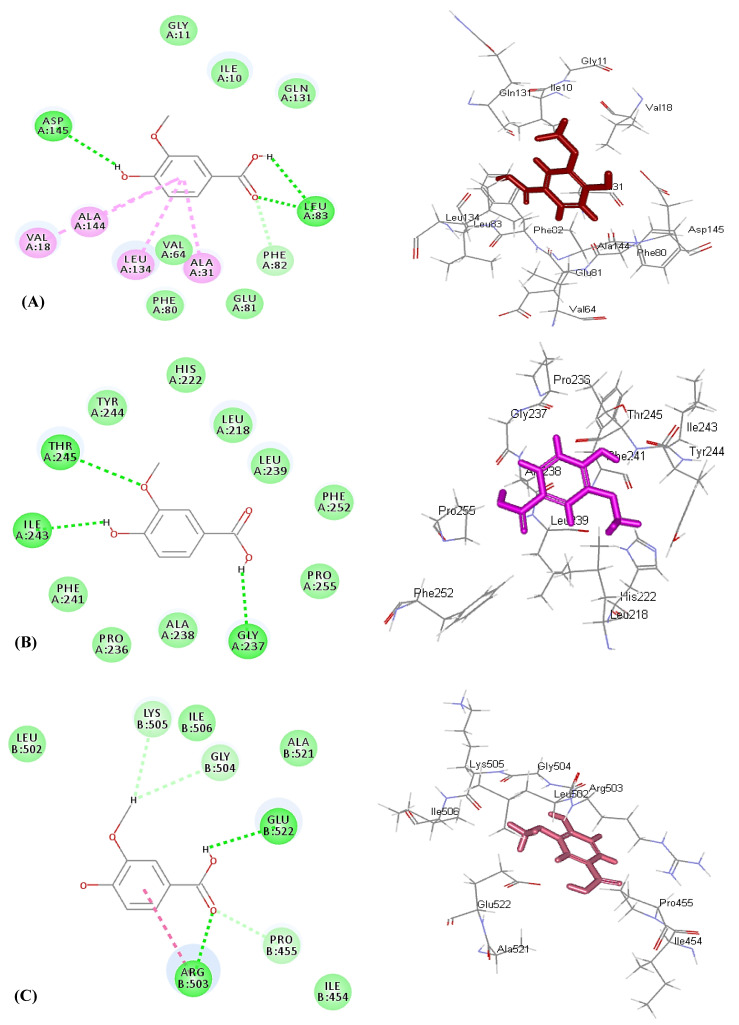
Two-dimensional and 3D binding mode of vanillic acid (**2**) within the active site of cyclin-dependent kinase 2 (**A**)**,** matrix metalloproteinase 13 (**B**), and DNA topoisomerase II (**C**).

Concerning enzymes involved in the occurrence and dissemination of cancerous cells, vanillic acid effectively inhibited both human cyclin-dependent kinase 2 (CDK-2) and DNA topoisomerase II with a high fitting score estimated by ∆G of −30.22 and −22.07 kcal/mole, respectively, approaching the co-crystalized ligand for the former (∆G = −39.34 kcal/mole) and exceeding that of the latter (∆G = −0.2 kcal/mole). However, vanillic acid showed moderate inhibition to matrix metalloproteinase 13 (MMP-13), displaying ∆G of −29.34 kcal/mole; meanwhile, MMP-13 co-crystallized ligand revealed ∆G of −72.33 kcal/mol; Table 3). Vanillic acid forms three H–bonds with Asp145 and Leu83, in addition to one C–H bond with Phe82 and four π–alkyl bonds with Val18, Ala144, Leu134, and Ala31 amino acid residues, at the active site of human cyclin-dependent kinase 2 (Figure 4A). Metalloproteinase 13 forms three H–bonds with Thr245, Ile243, and Gly237 (Figure 4B). Meanwhile, it forms two H–bonds with Arg503 and Glu237, three C–H bonds with Lys505, Gly504, and Pro455, and one amide-π bond with DNA topoisomerase II (Figure 4C). Thus, it is evident that vanillic acid exhibited its anticancer potential via inhibition of human cyclin-dependent kinase 2 that constitutes a group of enzymes that perfectly regulate progression and transcription of the cell cycle, arresting cell proliferation at G2/M-phase. In addition, it prohibited matrix metalloproteinase 13 that plays a crucial role in the degradation of the extracellular matrix of vital components that are important for the growth of malignant cells and responsible for their invasiveness, angiogenesis, and metastasis [37].

#### 2.3.2. ADME/TOPAKT Prediction

**Figure 5:**
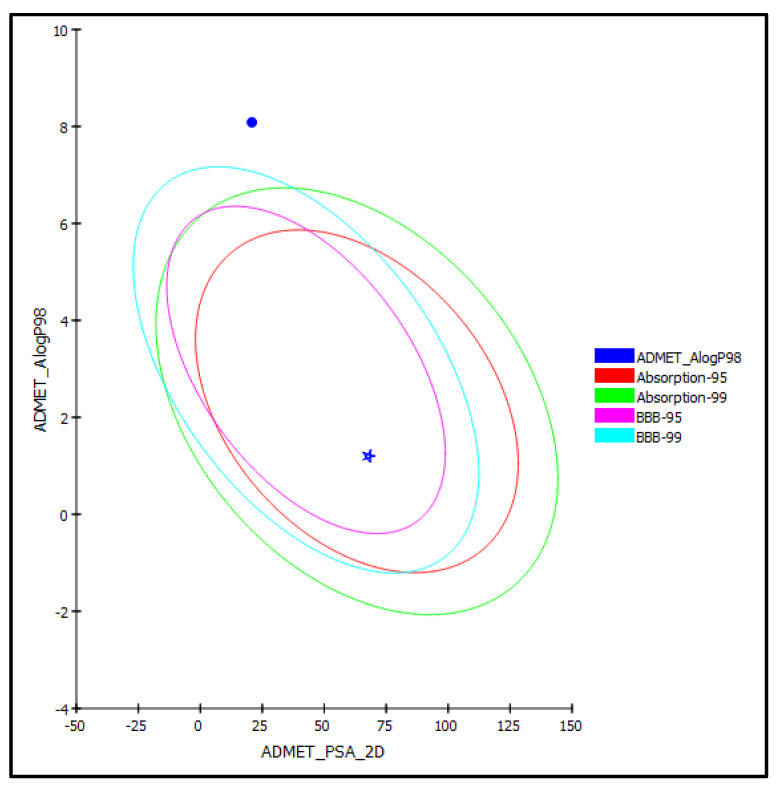
ADMET Plot for β-sitosterol (**1**) (circle) and vanillic acid (**2**) (star) isolated from *D. bupleuroides* displaying 95% and 99% confidence limit ellipses corresponding to the blood-brain barrier (BBB) and the human intestinal absorption models in ADMET_AlogP98.

β-Sitosterol (**1**) and vanillic acid (**2**) isolated from *D. bupleuroides* were exposed to AMET determination (absorption, distribution, metabolism, excretion, and toxicity), as well as toxicity evaluation (TOPKAT), to predict their pharmacodynamic, pharmacokinetic, and toxicity properties. Regarding AMET determination, vanillic acid (**2**) displayed less than 90% plasma protein binding and good human intestinal absorption with optimal solubility and low BBB penetration level. Thus, it lies within the 95% absorption ellipse, as illustrated in the ADMET plot (Figure 5). In contrast, β-sitosterol showed more than 90% plasma protein binding, very low human intestinal absorption, extremely low solubility, and undefined BBB penetration and, thus, lay outside 99% absorption ellipse. Furthermore, both compounds displayed no hepatotoxicity and caused no inhibition to cytochrome P450 2D6 (Table 4). Concerning TOPAKT prediction, both compounds showed no mutagenicity in the chemical *Ames* mutagenicity test; rat oral LD50 equals 1.57 and 2.39 g/kg.bw for β-sitosterol and vanillic acid, respectively, and chronic rat LOAEL of 0.002 and 0.19 g/kg.bw, respectively. Both compounds showed to be non-carcinogenic towards male NTP rats, but vanillic acid showed certain carcinogenicity towards female NTP rats. β-Sitosterol showed moderate skin irritancy with no irritation to the eye compared to vanillic acid that displayed moderate ocular irritancy with no irritation to the skin. Thus, that vanillic acid exhibited reasonable pharmacodynamic, pharmacokinetic, and toxicity properties and, therefore, could be incorporated in pharmaceutical preparations to counteract cancer and microbial invasion, as well as oxidative stress.

## 3. Materials and Methods

### 3.1. Plant Material

The whole plant of *Dicliptera bupleuroides* Nees (Acanthaceae family) was collected from Bhimber (Shamani), Kotli, Azad Kashmir in March and April 2016. The authentication and identification of the collected specimens were conducted by Dr. Uzma Hanif, Taxonomist of Botany Department, Government College University, Lahore, Pakistan, and are maintained in Department of Pharmacy, Punjab University College of Pharmacy, University of the Punjab, Lahore, Pakistan (voucher No: GC. Herb- Bot.3402). Specimens were powdered after being dried under shade for a suitable time. The powder was then stored in a cool, dry place till further studies.

### 3.2. General Experimental Procedure

The solvents including methanol, *n*-hexane, ethanol, chloroform, ethyl acetate, *n*-butanol, acetone, and petroleum ether were obtained from Merck, KGaA, Darmstadt, Germany. Besides, nitric acid, sulphuric acid, hydrochloric acid, and perchloric acid were purchased from (Sigma Aldrich Chemical, Steinheim, Germany). Aluminium-coated plates for TLC (silica gel 60 F254) and silica gel with mesh size 230–400 for column chromatography were purchased from Merck, KGaA, Darmstadt, Germany. NMR spectral analyses were done using AZ-300, Bruker spectrometers, Fällanden, Switzerland), FTIR analyses were performed using PerkinElmer Spectrum 3 Tri-Range FT-IR Spectrometer, Llantrisant, United Kingdom, whereas HPLC measurements were done using (Model LC-10AT, Shimadzu, Kyoto, Japan). The melting point is measured using the melting point apparatus (Mettler Toledo, Greifensee, Switzerland). The used rotary vacuum evaporator was Heidolph, model Laborata 4000, Schwabach, Germany; meanwhile, a UV lamp 254 and 365 nm (Fisher Scientific Ltd., Pandan Crescent, UE Tech Park, Singapore) was used for UV detection, while a UV/Visible spectrophotometer (Hitachi-UV-3200, Tokyo, Japan) was used for UV measurements.

### 3.3. Extraction and Isolation

First, 5 Kg of shade-dried powdered plant material were accurately weighed and macerated in 6 L of methanol at room temperature for one week using the cold maceration method. The filtration was performed for the methanol extract and was repeated three times (6 L × 3). The collected filtrate was dried by using a rotary evaporator at 40–45 °C under reduced pressure and temperature not exceeding 45 °C to obtain 340 g of semisolid residue (DBT). Then, this residue was mixed with water and consequently fractionated using different solvents of increasing order of polarity, namely *n*-hexane, chloroform, ethyl acetate, and *n*-butanol). The separation of the main compounds from the chloroform fraction (DBC) and ethyl acetate fraction (DBE) was achieved by using column chromatography where 300 g silica gel (mesh size 230–400) were used to fill a glass column with dimensions 50 cm in height and 2 cm in diameter. The application of 10 g DBC was performed on the silica gel adopting dry loading method, and separation was started by using *n*-hexane as a mobile phase followed by increasing polarity and finally washed with methanol to give 80–90 vials. TLC was used to monitor similar fractions under a UV lamp of λ of 254 nm that were collected together based upon R_f_ values and appearance, where the elution by the mobile phase *n*-hexane: chloroform (95:05) resulted in the isolation of compound (**1**) (22 mg). A similar procedure was adopted for (DBE) that resulted in the isolation of compound (**2**) (15 mg) upon elution of the silica gel column with mobile phase *n*-hexane: chloroform (80:20).

### 3.4. Compounds Characterization

#### 3.4.1. Characterization of Compound (1)

Compound (**1**) was collected as white needle-like crystals. Melting point is 138 °C. IR spectrum (V max; MeOH) of compound (**1**) revealed the presence of band peaks at 2919.97, 2851.15, 1707.93, 1463.11, 1377.05, 1287.76, 1228.56, 1189.61, 1051.19, 936.30, 839.21, 721.66, 686.32, and 609.40 cm^−1^. ^1^H-NMR (500 MHz, CDCL_3_) δ: 5.34 (1H, m, H-6), 2.34 (1H, m, H-3), 1.16 (1H, s, H-2), 0.98 (1H, s, H-1), 0.66 (3H, m, 18-CH_3_, 19-CH_3_). DEPT (135 MHz, CDCL_3_) δ: 36.12 (C-1), 29.77 (C-2), 71.85 (C-3), 42.27 (C-4), 138.32 (C-5), 121.75 (C-6), 32.97 (C-8), 50.14 (C-9), 36.15 (C-10), 21.03 (C-11), 37.25 (C-12), 45.85 (C-13), 56.78 (C-14), 23.07 (C-15), 26.78 (C-16), 56.78 (C-17), 11.98 (C-18), 19.40 (C-19), 33.81 (C-20), 19.03 (C-21), 32.05 (C-22), 24.29 (C-23), 50.14 (C-24), 29.03 (C-25), 19.82 (C-26), 19.40 (C-27), 21.09 (C-28), 14.06 (C-29) [15]. ^1^H-NMR and ^13^C-NMR and IR spectral data of compound (**1**) are represented as Figure S1 in the Supplementary Materials.

#### 3.4.2. Characterization of Compound (2)

Compound (**2**) was obtained as white solid. Melting point is 210–213 °C. IR spectrum of compound (**2**) spectrum (V max; MeOH) revealed the presence of band peaks at 3479, 2836, 1670, 1594, 1521, 1472,1454, 1433, 1376, 1276, 1235, 1201, 1108, 1026, 915, 880, 818,805, 756, 721, and 636 cm^−1^. ^1^H-NMR (500MHz, CDCl_3_) δ: 7.70 (1H, dd, *J* = 1.5, 2.0 Hz, H-2), 7.57 (1H, d, *J* = 2.0 Hz, H-6), 6.04 (1H, s, H-5), 3.94 (3H, s, OCH_3_) [20,21]. ^1^H-NMR and IR spectral data of compound (**2**) are represented as Figure S2 in the Supplementary Materials.

### 3.5. In Vitro Biological Evaluation of D. bupleuroides Isolated Compounds

#### 3.5.1. In Vitro Antioxidant Activity Evaluation Using 1,1-Diphenyl-2-Picrylhydrazyl (DPPH*) Radical Scavenging Capacity Assay

Antioxidant activity of *D. bupleuroides* isolated compounds was determined using 1,1-Diphenyl-2-Picrylhydrazyl (DPPH*) radical scavenging capacity assay [22]. Stock solutions of the isolated compounds in methanol in the concentration of 1 mg/mL were prepared methanol. Similarly the standard ascorbic acid was also prepared in a concentration of 1 mg/1 mL.Meanwhile, pure methanol was used as a control. Different concentrations (50, 100, 150, 200, and 250 μg/mL) of purified compounds and ascorbic acid (standard compound) were prepared. All samples and the standard were incubated at room temperature for 30 min, and then the absorbance was recorded at λ = 517 nm by a UV spectrophotometer. This assay was repeated in triplicates. The percentage of inhibition IC_50_ was calculated according to the following equation:

[Ac − As/Ac] × 100(1)

where Ac: absorbance of control; As: absorbance of the sample [38].

#### 3.5.2. In Vitro Antimicrobial Activity Evaluation Using Disc Diffusion Assay

The isolated compounds were studied for their antimicrobial effect using the disc diffusion assay method against a panel of bacteria and fungi, namely *Staphylococcus aureus* (ATCC 6538), *Bacillus substilis* (ATCC 6633), *Echercheria coli* (ATCC 8739), *Pseudomonas aeruginosa* (ATCC 9027) and fungal strains *Candida albicans* (ATCC 10231) and *Penicillium notatum* (ATCC 11709), obtained from Pacific Pharmaceutical LTD, Lahore, Pakistan [39]. A sterilized filter paper in the form disc was saturated with the measured quantity (10 μL) of the sample with a 0.5 mg/mL concentration. It was positioned in a plate of 7 cm diameter having bacterial or fungal medium seeded with the spore suspension of the test microorganism. The next day after incubation at 37 °C for bacteria and 25 °C for three days for fungi, the diameter of the inhibition zone surrounding the sample is taken to measure the inhibitory power of the compounds against the particular test organism. Commercial ciprofloxacin and fluconazole were used as reference drugs for broad-spectrum antibacterial and antifungal agents, respectively. All these steps were done under sterile conditions. The test was repeated in triplicates.

#### 3.5.3. In Vitro Anticancer Activity Evaluation Using MTT (3-(4,5-dimethylthiazol-2-yl)- 2,5-Diphenyl-2H-Tetrazolium Bromide) Assay

This assay was used to determine the anticancer potential of *D. bupleuroides* isolated compounds adopting the method previously described by Mosmann et al., in 1983, with certain modifications [40]. Briefly, a 96-well microplate was seeded with 200 µL suspension of Hep G2 cells and then was incubated at 37 °C in a CO_2_ (5%) environment for 24 h at 95% humidity. After incubation, cells were observed under a microscope without disturbing them; settled down at the bottom of the microplate. Then, these cells were exposed to different concentrations of each sample; 200 µL of each stock solution were added in a labeled microplate well and incubated again for 24 h in similar conditions. After incubation, 20 µL of MTT reagent (5 mg/mL in DMSO) was added to the wells and was incubated again for 4 h. Next, 150 µL of DMSO were added in each well. Then, the formazan crystal was dissolved and gave purple color with MTT reagent. After 20 min of incubation, the absorbance was recorded at λ = 600 nm at the ELISA reader and compared with positive and negative control [41,42]. Percentage viability of cell was calculated as

% Cell viability = Absorbance of treated cells/ Absorbance of controlled cells × 100%.(2)

#### 3.5.4. Statistical Analysis

All experiments were done in triplicate, and the results were represented as mean ± SD (standard deviation). Graphs are drawn using GraphPad Prism 5 (GraphPad Software, Inc., San Diego, CA, USA).

### 3.6. In Silico Studies

#### 3.6.1. Molecular Docking Experiments

Molecular docking was performed for compounds (**1**) and (**2**) using Discovery Studio 4.5 (Accelrys Inc., San Diego, CA, USA) with C-docker protocol on different proteins that are crucial in the occurrence and dissemination of microbial infection and cancerous cells. They are DNA-gyrase (PDB ID 4Z2D; 3.38 Å); dihydrofolate reductase (PDB ID 4KM2; 1.4 Å); aminoglycoside nucleotidyltransferase (PDB ID 4WQL; 1.73 Å); and β-lactamase (PDB ID 3NBL; 2.0 Å) for antimicrobial assessment and human cyclin-dependent kinase 2(CDK-2) (PDB ID 1PXP, 2.30 Å), matrix metalloproteinase 13 (MMP-13) (PDB ID 1XUD, 1.8Å); and human DNA topoisomerase II (TOP-2) (PDB ID 4G0U, 2.70 Å) for anticancer evaluation obtained from the protein data bank (www.pdb.org, (accessed on 17 October 2021)), following what was previously reported [43,44].

#### 3.6.2. ADME/TOPAKT Prediction

Herein, compounds (**1**–**2**) isolated from *D. bupleuroides* were exposed to AMET determination (absorption, distribution, metabolism, excretion, and toxicity), as well as toxicity evaluation (TOPKAT), adopting Discovery Studio 4.5 (Accelrys Inc., San Diego, CA, USA) to determine their pharmacodynamic, pharmacokinetic, and toxicity properties. The selected descriptors for ADMET prediction are aqueous solubility, Blood-brain barrier penetration (BBB), cytochrome P450 2D6, human intestinal absorption (HIA), plasma protein binding prediction (PPB), and hepatotoxicity level. However, the chosen TOPKAT parameters were skin and ocular irritation, Ames mutagenicity, rat oral LD50, and rat Chronic LOAEL, as well as the carcinogenic effect on male and female rat NPT (National Toxicology Program) [45].

## 4. Conclusions

Phytochemical investigations of the chloroform and ethyl acetate fractions of *D. bupleuroides* resulted in the isolation of β-sitosterol from the former, whereas vanillic acid from the latter reported for the first time from this plant species. Both compounds revealed remarkable antioxidant potential; meanwhile, vanillic acid exerted considerable antimicrobial and anticancer activity, as evidenced by the in vitro studies. These results were further consolidated by in silico molecular modeling studies on different enzymes. Additionally, ADME/TOPKAT prediction showed that vanillic acid exhibited reasonable pharmacodynamic, pharmacokinetic, and toxicity properties and, thus, could perfectly, together with *D. bupleuroides*, be incorporated in pharmaceutical preparations to counteract cancer and microbial invasion, as well as oxidative stress. Thus, it is concluded that *D. bupleroides* could be a potential source of therapeutically active compounds, which would be helpful for the discovery of clinically effective and safe drugs. However, further in-depth in vivo studies performed on animal models, followed by preclinical trials, should support the obtained results.

## Figures and Tables

**Table 1:** In vitro antioxidant activity evaluation for β-sitosterol (**1**) and vanillic acid (**2**) isolated from *D. bupleuroides* using 1,1-diphenyl-2-picrylhydrazyl (DPPH*) radical scavenging capacity assay.

Sample	IC_50_ (µg/mL)
β-Sitosterol (**1**)	198.87
Vanillic acid (**2**)	92.68
Ascorbic acid	125.86

% inhibition is measured in triplicates (*n* = 3) and presented as Mean ± SD. * Significantly different from the ascorbic acid group at *p* < 0.05.

**Table 2:** MIC values of β-sitosterol (**1**) and vanillic acid (**2**) isolated from *D. bupleuroides* using disc diffusion assay and expressed in mg/mL.

Microorganism	β-Sitosterol (1)	Vanillic Acid (2)	Ciprofloxacin	Fluconazole
*S. aureus*	0.809	0.529	0.08	NT
*P. aeruginosa*	0.700	0.486	0.05	NT
*B. subtilis*	0.599	0.467	0.04	NT
*E. coli*	0.672	0.492	0.06	NT
*C. Albicans*	0.182	0.02	NT	0.12
*P. notatum*	0.001	0.001	NT	0.02

NT: not tested.

**Table 3:** Binding energies (kcal/mole) of β-sitosterol (**1**) and vanillic acid (**2**) isolated from *D. bupleuroides* within the active sites of crucial microbial and cancer triggering enzymes.

Examined Enzymes	β-Sitosterol	No of H–Bonds	Vanillic Acid	No of H–Bonds	Co-Crystalized Ligands	No of H–Bonds
Microbial Enzymes						
DNA-gyrase	56.45	-	−24.26	1; Glu474	−9.7	2; Asp435, Ser436
Dihydrofolate reductase	37.92	1; Arg32	−26.63	2; Ile94, Asp27	−28.90	3; Ile94, Ala7
Aminoglycoside nucleotidyltransferase	42.37	1; Glu101	−25.21	2; Asp46, Asp86	−20.03	8; Glu138, Asp86, Gly27, Leu47, Thr48
β-Lactamase	37.64	-	−25.10	3; Ile117, Lys87, Ser84	−61.76	7; Ile117, Lys87, Ser84, Ser142, Thr253, Lys250
Anticancer Enzymes						
Cyclin-dependent kinase 2	44.74	-	−30.22	3; Asp145, Leu83	−39.34	1; Leu83
Matrix metalloproteinase 13	93.47	1; Phe241	−29.34	3; Thr245, Ile243, Gly237	−72.33	4; Thr245, Lys140, Thr247, Ala238
DNA topoisomerase II	53.85	-	−22.07	2; Arg503, Glu237	−0.2	-

The co-crystalized ligands are levofloxacin for DNA-gyrase; trimethoprim for dihydrofolate reductase; kanamycin for aminoglycoside nucleotidyltransferase; cefuroxime for β-lactamase; CK8, N-[4-(2,4-dimethyl-thiazol-5-yl)-pyrimidin-2-yl]-N′, N′-dimethyl benzene-1,4-diamine; for cyclin-dependent kinase 2; PB4, N, N′-bis (4-fluoro-3-methylbenzyl) pyrimidine-4,6-dicarboxamid for matrix metalloproteinase 13; doxorubicin for DNA topoisomerase II.

**Table 4:** ADME/TOPKAT (absorption, distribution, metabolism, excretion, and toxicity) evaluation of β-sitosterol (**1**) and vanillic acid (**2**) isolated from *D. bupleuroides*.

Compounds	β-Sitosterol (1)	Vanillic Acid (2)
ADMET parameters		
Absorption Level	3	0
Solubility Level	0	4
BBB Level	4	3
PPB Level	True	False
CPY2D6	NI	NI
Hepatotoxic	Non-toxic	Non-toxic
PSA-2D	8.08	1.20
Alog p98	20.82	67.82
TOPKAT parameters		
Ames prediction	Non-mutagen	Non-mutagen
Rat chronic LOAEL (g/kg.bw)	0.002	0.19
Rat oral LD50 (g/kg.bw)	1.57	2.39
Rat female NPT	Non-carcinogen	Carcinogen
Rat Male NPT	Non-carcinogen	Non-carcinogen
Skin irritancy	Moderate	None
Ocular irritancy	None	Moderate

0, 1, 2, and 3 indicates good, moderate, low, and very low absorption, respectively; 0, 1, 2, 3, 4, and 5 indicates extremely low, very low but possible, low, good, optimal, and too soluble, respectively; 0, 1, 2, 3, and 4 denote very high, high, medium, low, and undefined, penetration via BBB, respectively; PBB, plasma protein binding, FALSE means less than 90%, TRUE means more than 90%; NI: Non-inhibitor.

## Data Availability

Data are available upon request from the authors.

## Supplementary Materials

Figure S1: ^1^H NMR (A), DEPT-90 (B), DEPT-135 (C), and IR (D) spectra of β-sitosterol (**1**), Figure S2: ^1^H NMR (A) and IR (B) spectra of vanillic acid (**2**)**,** Figure S3: 2D and 3D binding mode of vanillic acid (**2**) within the active site of β-lactamase
