# Supplementary material for: Evaluation of The Antioxidant, Antimicrobial, and Anticancer Activities of Dicliptera bupleuroides Isolated Compounds Using In Vitro and In Silico Studies
Source: Molecules. 2021 Nov 27;26(23):7196. doi: 10.3390/molecules26237196 (PMC8659019; doi:10.3390/molecules26237196)
Supplement: Supplementary file 1 [file molecules-26-07196-s001.zip › molecules-1466372-SP.pdf]

Evaluation of the antioxidant, antimicrobial and anticancer activity of *Dicliptera bupleuroides* isolated compounds using *in vitro* and *in silico* studies

Supplementary materials

Figure S1: ^{1}H NMR (A), DEPT-90 (B), DEPT-135 (C) and IR (D) spectra of β-sitosterol **(1)**.

Figure S2: ^{1}H NMR (A) and IR (B) spectra of vanillic acid **(2)**

Figure S3: 2D and 3D binding mode of vanillic acid (2) within the active site of β-lactamase

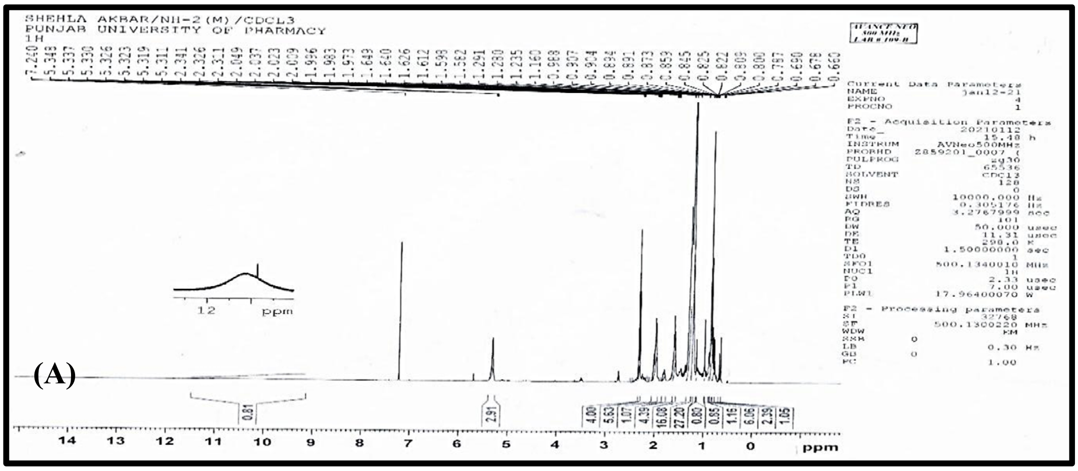

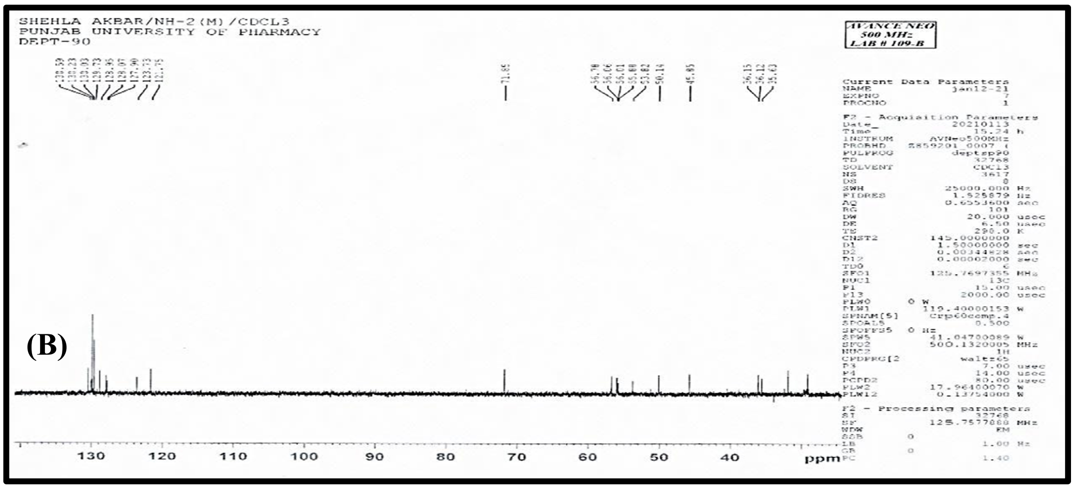

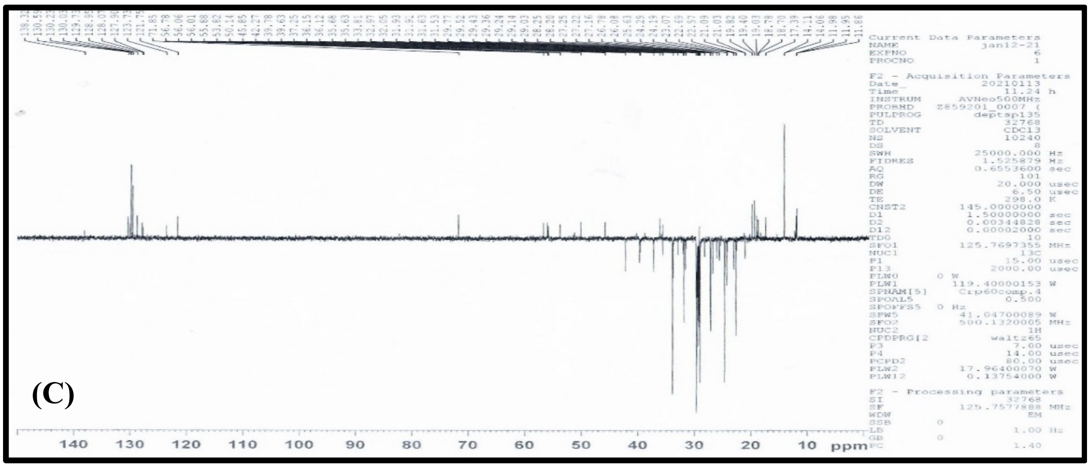

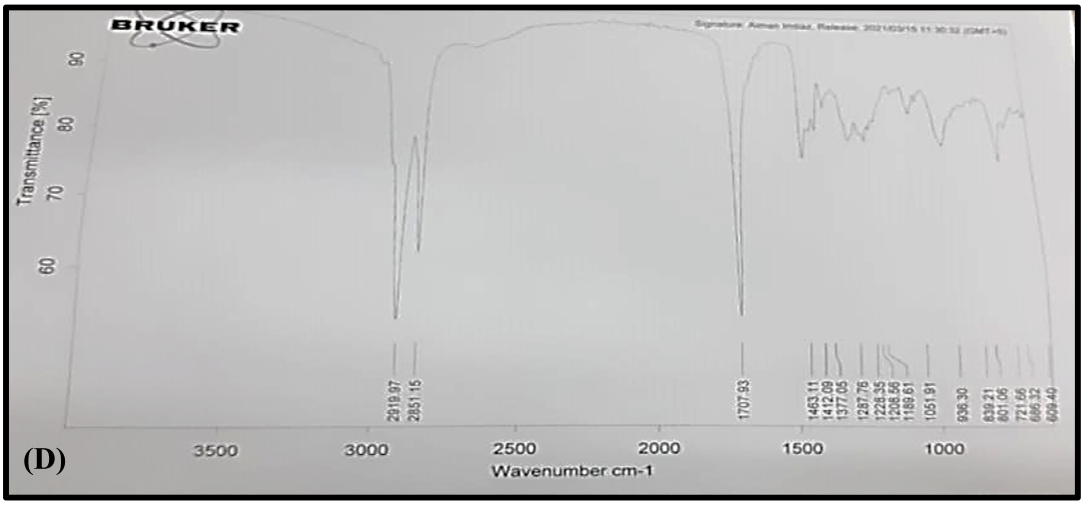

Figure S1 ^{1}H NMR (A), DEPT-90 (B), DEPT-135 (C) and IR (D) spectra of *β*-sitosterol **(1)**.

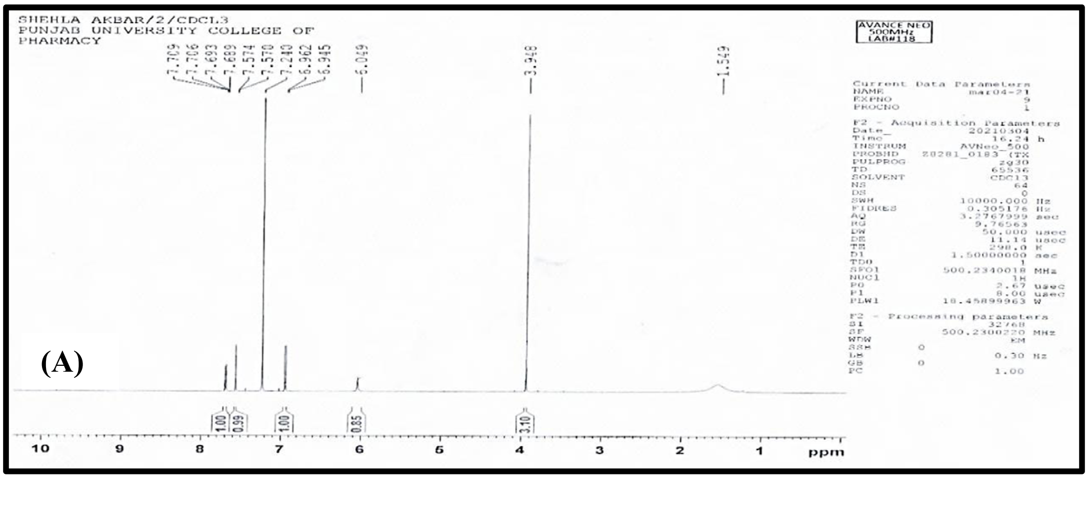

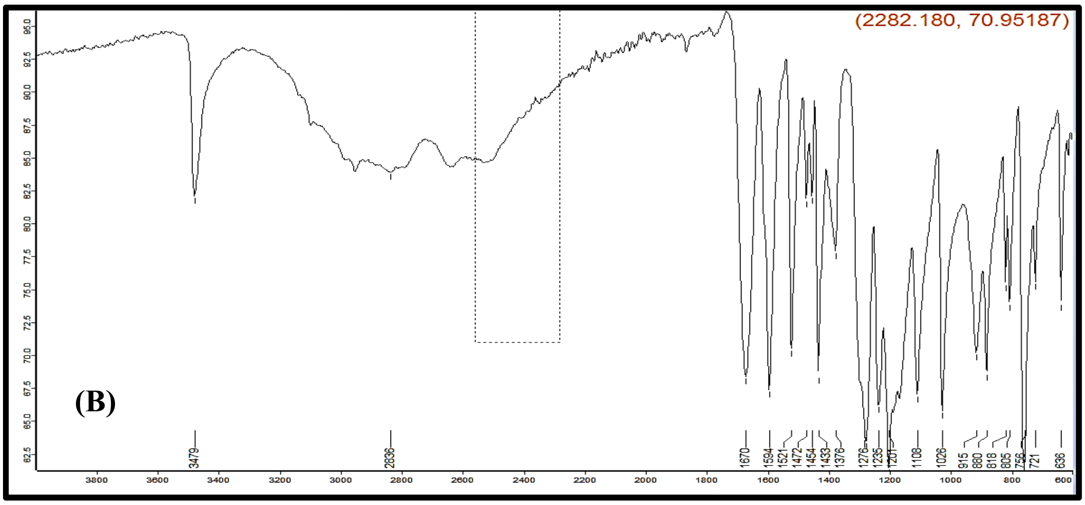

Figure S2 ^{1}H NMR (A) and IR (B) spectra of vanallic acid **(2)**

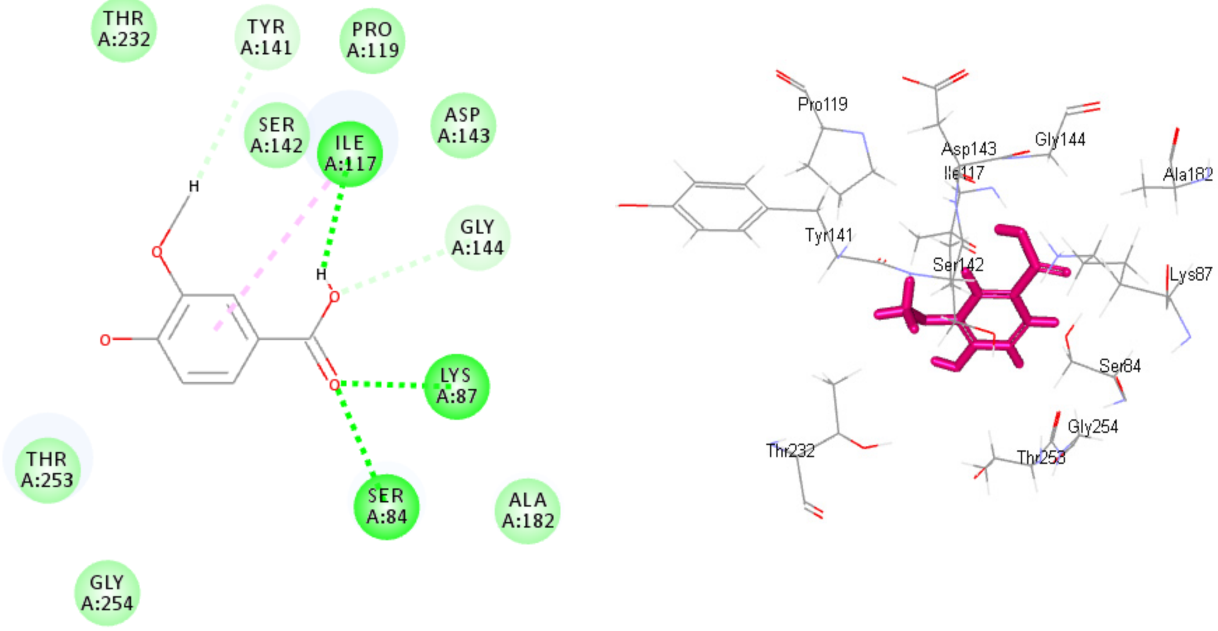

Figure S3: 2D and 3D binding mode of vanillic acid (2) within the active site of *β*-lactamase
